# Recent Insights into the Role of PPARs in Disease

**DOI:** 10.3390/cells12121572

**Published:** 2023-06-07

**Authors:** Nicole Wagner, Kay-Dietrich Wagner

**Affiliations:** CNRS, INSERM, iBV, Université Côte d’Azur, 06107 Nice, France

Peroxisome proliferator-activated receptors (PPARs) are nuclear receptors that play important roles in cell proliferation, differentiation, metabolism, and cancer [1,2,3,4,5]. They were originally identified more than 30 years ago [6,7] in a search for the receptors for a group of rodent hepatocarcinogens that cause the proliferation of peroxisomes. To look on the bright side of life, one of these rodent hepatocarcinogens (clofibrate) was also known to lower triglycerides and cholesterol concentrations in the plasma of patients and to be beneficial in the prevention of ischemic heart disease in a population with increased plasma cholesterol levels [6]. These effects were well known long before the cloning of the corresponding PPARs as receptors [8]. It was also known that these drugs “coincidently” induce an increased transcription of genes required for the peroxisomal β-oxidation of long-chain fatty acids and genes of the cytochrome P450 family [6,9,10,11]. Shortly after, it was realized that these receptors not only somehow induce genes of fatty acid metabolisms but are also activated by fatty acids [12]. After these first timid steps and with the increasingly powerful tools of modern mouse genetics and molecular and cellular biology methods, our knowledge about PPARs is increasing exponentially. Today, basic knowledge about the three different isoforms, PPARα, PPARβ/δ, and PPARγ, is well-established, and PPARα and PPARγ agonists have been in clinical use for a long time for the treatment of hyperlipidemia and type 2 diabetes, respectively. Nevertheless, the topic of PPARs attracts a lot of attention, and after a first successful Special Issue titled “The Role of PPARs in Disease” in *Cells* in 2020 [1], we decided to collect novel, exiting data and highly interesting points of view in the form of original articles and reviews for the current Special Issue, titled “The Role of PPARs in Disease II”. Here, we will briefly outline and highlight the most recent insights into the roles of PPARs in disease collected in this Special Issue.

Steinke et al. describe a novel PPARβ/δ and PPARγ dual agonist, which demonstrates striking beneficial effects in a mouse model (3xTgAD) of Alzheimer’s disease [13]. PPARγ agonists had been tested for this indication already before in several studies, but the effects were limited due to the poor penetration of the blood–brain barrier requiring high doses and observed severe side effects in clinical trials [14,15,16]. As PPARβ/δ is highly expressed in the brain compared to other isoforms and PPARβ/δ activation might counteract weight gain, the authors reasoned that a dual agonist might have additional beneficial effects compared to the PPARγ agonists reported before. They showed first that their compound AU9 activates PPARγ and PPARβ/δ. Most importantly, AU9 improves memory deficits in 3xTgAD mice, improves neurotrophin expression and spine density, reduces amyloid beta levels in the brain, and diminishes neuroinflammation. In contrast to the PPARγ agonist pioglitazone, the novel dual agonist caused less weight gain and heart hypertrophy but was still able to reduce blood glucose levels in 3xTgAD transgenic mice. Given this exiting profile of action, this novel dual agonist might represent great promises for people suffering from Alzheimer’s disease. Future experiments will show whether PPARβ/δ activation by the compound is also angiogenic, as reported for other models of PPARβ/δ stimulation [17,18,19,20,21] and if this novel therapeutic approach for Alzheimer’s disease is safe in the settings of cancer and ocular disease.

Melissa Rayner and colleagues explored in their paper the utility of several compounds with PPARγ agonist activity in a different setting of neurological disease, i.e., peripheral nerve injury (PNI) [22]. The regeneration and remyelination of damaged nerves are essential for functional recovery, but only little progress had been made in clinical settings to stimulate repair. PPARγ acts as an inhibitor of the Rho/ROCK pathway. This inhibition enhances axon regeneration after PNI [23], making PPARγ a good candidate to stimulate peripheral nerve regeneration. Non-steroidal anti-inflammatory drugs (NSAIDs) and thiazolidinediones have shown beneficial effects on nerve regeneration, which seems to be mediated via their PPARγ agonist activity [24]. The authors combined in vitro co-culture systems of nerve and Schwann cells and in vivo models of axon regeneration in rats to explore whether the potency of different molecules to induce nerve regeneration in vitro and in vivo corresponds to their PPARγ agonist activity. All tested molecules with PPARγ agonist activity promoted to some extend neuronal outgrowth in vitro, but functional tests in the animals treated in vivo showed no significant differences. Thus, it is possible that additional mechanisms, e.g., PPARγ effects on immune system function, might modify the outcomes in the in vivo versus in vitro situation. It might be an interesting future approach to compare existing clinical data from patients with PNI treated or not treated with different NSAIDs or PPARγ agonists to answer the question of whether they might experience a functional benefit from different PPARγ agonist treatment. Of note, these analyses should be conducted by taking into account possible gender differences as it has emerged, for instance, that pioglitazone produces a female-predominant inhibition of hyperalgesia associated with peripheral nerve injury in rodents [25].

Tomczyk and colleagues report that the PPARγ agonist rosiglitazone improves cardiac and muscle function in a mouse model of Huntington’s disease [26]. Huntington’s disease is a rare genetic disease affecting the central nervous system, but it also negatively impacts the heart and muscle strength. In a non-diabetic Huntington’s disease mouse model, the authors found that treatment with rosiglitazone improves grip strength and cardiac contractile function. These functional improvements were accompanied by an enhancement of the total adenine nucleotides pool, increased glucose oxidation, alterations in mitochondria number and function, and increased total antioxidant status. As heart failure is a frequent cause of death in Huntington’s disease patients, it would be highly exciting if similar results could be observed in clinical studies. Additionally, already available data from patients with Huntington’s disease might be re-analyzed for a potential medication with PPARγ agonists, which were in use for a long time for the treatment of diabetes.

Papaccio et al. explored another potential use of the PPARγ agonist pioglitazone. They treated vitiligo melanocytes and fibroblasts with pioglitazone and reported increased mRNA and protein levels of anaerobic glycolytic enzymes, restored mitochondrial membrane potential and mitochondrial DNA (mtDNA) copy number, an increase in ATP content and a decrease in reactive oxygen species (ROS) production, and a reversal of a premature senescence phenotype in vitiligo melanocytes [27]. As the current treatment options for vitiligo, an acquired pigmentation disorder of the skin, are limited, the potential use of PPARγ agonists might represent a novel therapeutic opportunity. Future clinical studies will clarify whether vitiligo patients could benefit from this alternative treatment strategy.

Grimaldi et al. explored potential beneficial effects of the PPARγ agonist rosiglitazone on the angiogenic profile of preeclampsia (PE) placentas [28]. PE is one of the most common causes of maternal-fetal morbidity and mortality. Placentas in PE are characterized by reduced PPARγ expression, disturbed trophoblast differentiation, and the abnormal secretion of angiogenic factors, which causes systemic endothelial damage and organ dysfunction. Thus, the idea of activating PPARγ to induce the normalization of these alterations seems to be straightforward. The authors cultured normal and PE placenta tissue in the presence or absence of rosiglitazone and used cell culture supernatants to characterize angiogenic properties in human umbilical vein endothelial cell (HUVEC) tube formation assays. They showed beneficial effects of rosiglitazone treatment on the angiogenic profile in the human preeclamptic placenta through a reduction in anti-angiogenic angiopoietin-2 and soluble endoglin and the upregulation of pro-angiogenic placental growth factor, fibroblast growth factor-2, heparin-binding epidermal growth factor, and follistatin. The treatment of PE placental tissue with the PPARγ agonist enhanced the angiogenic profile of HUVECs exposed to the cell culture supernatant. Thus, it will be highly interesting to see in future studies whether rosiglitazone represents a therapeutic opportunity for PE. Besides this original investigation, the role of PPARs in PE has been reviewed recently [29].

Li and colleagues also investigated the role and potential therapeutic opportunities of PPARγ activation in the placenta in a different context [30]. It is known that exposure to the antibacterial agent triclosan (TCS), which acts also as endocrine disruptor, results in placental abnormalities, increased abortion rates, and the reduced size of fetuses and newborns. The authors show that TCS downregulates the expression of PPARγ and its downstream genes HMOX1, ANGPTL4, VEGFA, MMP-2, and MMP-9 and upregulates inflammatory genes p65, IL-6, IL-1β, and TNF-α in vitro and in vivo. The overexpression of PPARγ or activation of the receptor by rosiglitazone improved cell viability, migration, and angiogenesis and reduced the inflammatory response caused by TCS. The knockdown or inhibition of PPARγ had the opposite effects. Finally, TCS caused placenta dysfunction characterized by a significant decrease in the weight and size of the placenta and fetus, while the PPARγ agonist rosiglitazone reduced this damage in mice. Hopefully, in the future, we will be able to reduce industrial pollution with endocrine disruptors instead of treating the damage with PPARγ agonists.

From in vitro studies and pre-clinical animal models in vivo, it has been known that PPARγ activation protects kidney podocytes from injury and reduces proteinuria and glomerular diseases (reviewed in [31]). However, PPARγ signaling in podocytes seems to be different from its well-understood role in driving insulin sensitivity and adipogenesis. Bryant et al. showed in this Special Issue of *Cells* that the expression of PPARγ splice variants differ between podocytes and adipocytes and liver [32]. Podocytes express the PPARγ Var 1 (encoding γ1) but not γ2, which is expressed in adipocytes. Low levels of PPARγ Var4, Var3, Var11, VartORF4, and Var9 were also detected in podocytes. Interestingly, a distinct podocyte vs. adipocyte PPAR-promoter response element was also identified in podocytes, which puts our concept of common PPAR-response element sequences in question. This study represents a rationale for the search of novel PPARγ splice-specific agonists, which could be highly specific for targeted therapies.

Besides the multiple roles of PPARγ activation, PPARα agonists also exert several functions in addition to lipid lowering [33]. Qiu and colleagues show that the activation of PPARα ameliorates cardiac fibrosis in Dsg2-deficient arrhythmogenic cardiomyopathy [34]. Arrhythmogenic cardiomyopathy (ACM) represents a genetic disease characterized by the progressive fibro-fatty replacement of cardiac myocytes. Mutations in desmoglein-2 (Dsg2) are one of the reasons for the development of ACM. The authors showed that cardiac-specific Dsg2 knockout mice develop fibrosis, have reduced PPARα levels, and increased STAT3 and SMAD3 activity. Fenofibrate treatment as well as viral PPARα overexpression improved cardiac fibrosis and decreased the phosphorylation of STAT3, SMAD3, and AKT in cardiac-specific Dsg2 knockout mice, suggesting a novel indication for the use of PPARα agonists in ACM patients.

Adamowicz et al. postulate in this Special Issue that hepatic PPARα is suppressed in primary biliary cholangitis, which might be modulated by miR-155 [35]. The authors show that PPARα expression is reduced in human biliary cholangitis samples compared to controls. Additionally, miR-155 and miR-21 were increased in the samples from patients with primary biliary cholangitis. In human hepatocarcinoma (HepG2) and normal human cholangiocyte (NHC) cells transfected with miR-155 or miR-21 mimics, the effect on PPARα was variable. Whether these microRNAs have a direct effect on PPARα expression and whether the reduction in PPARα in primary biliary cholangitis is causative for disease progression remains to be determined.

Non-alcoholic steatohepatitis (NASH) is a stage of non-alcoholic fatty liver disease (NAFLD) which might lead to fibrosis, liver cirrhosis, and carcinomas. The only established clinical treatment is bariatric surgery, but trials with PPAR agonists, i.e., the dual PPARα/β/δ agonist elafibranor for the treatment of NASH, were conducted [36]. In the current Special Issue, Boeckmans and colleagues compared the transcriptome profiles of in vitro NASH human cell culture models with cells treated with elafibranor. Additionally, they compared the elafibranor-induced gene expression modulation to the transcriptome data of patients with improved/resolved NAFLD/NASH upon bariatric surgery [37]. The authors found a 35% overlap of transcriptome data from NASH patients with cell culture models exposed to NASH-inducing triggers. Elafibranor partially reversed the transcriptional modulations. Peroxisome Proliferator Activated Receptor Alpha, PPARG Coactivator 1 Alpha, and Sirtuin 1 were the major common upstream regulators upon exposure to elafibranor. Angiopoietin-Like 4, pyruvate dehydrogenase kinase 4, and perilipin 2 were commonly upregulated by elafibranor in the in vitro NASH models but not in patient samples after bariatric surgery. These generated large datasets are very informative. They provide evidence for the differences in the in vitro models with the ex vivo patient data and also, not unexpectedly, a different response to the dual PPAR agonist and bariatric surgery. Still a major challenge for the use of the in vitro NASH models for large-scale drug screening remains the identification of a common robust marker set, which ideally would be easy to measure and analyze.

In a highly exciting study, Murakami et al. used a combination of the selective PPARα modulator pemafibrate and sodium-glucose cotransporter 2 inhibitor tofogliflozin to treat NASH in a mouse model [38]. They carefully investigated histopathological changes in NASH animals, mice with single compound treatment, and the combination of pemafibrate and tofogliflozin compared to control animals. The authors provide evidence that the combination effectively reduces hepatocyte degeneration and improves hypertriglyceridemia, hyperglycemia, and macro vesicular steatosis. Most importantly, the combination significantly reduced the number of tumors and improved survival in the mouse NASH model. Hopefully, clinical studies in the future will provide comparable results in human patients, which would represent a major breakthrough in the field.

In the reviews as part of the current Special Issue, Kim and colleagues summarized current knowledge about the potential involvement of the different PPARs in infectious diseases [39]. Although the involvement of PPARs in metabolism and inflammatory responses is well characterized, relatively little is known about the modulatory roles of PPARs in viral, bacterial, and parasitic infections. The authors carefully summarize the current knowledge in the field and introduce future perspectives as, currently, no PPAR therapeutics are in use to treat infectious diseases.

Zhao et al. reviewed the involvement of PPARs in breast cancer. They introduce the structure of the different PPARs and the mechanisms of PPAR-mediated gene regulation and provide examples for the structure of PPAR agonists and antagonists. Afterwards, they describe in detail the knowledge of each PPAR isotype in breast cancer, including multiple observations reported from different cell lines and a potential modulation of PPAR effects by estrogen receptors, which are of special importance in breast cancer and are an established therapeutic target.

Basilotta et al. explore the potential therapeutic effects of PPAR ligands in glioblastoma [40]. Glioblastoma is the most aggressive brain tumor, with very limited therapeutic options; thus, additional therapeutic strategies are urgently needed. PPARα and PPARγ activation are thought to inhibit tumor growth, while PPARβ/δ seems to be mostly pro-tumorigenic, although it might reduce the cardiotoxicity of doxorubicin used for glioblastoma treatment. The PPARα agonist fenofibrate has received the most attention due to its capacity to reduce the proliferation of glioblastoma cells through both PPAR-dependent and PPAR-independent mechanisms. PPAR-γ ligands have been reported to induce cell death in glioblastoma cells. Further studies are required to define the potential clinical use of PPAR modulators for glioblastoma.

Ballav and colleagues, in their review, focus on the utility of PPARγ activators for the treatment of cancer [41]. They introduce structure and functional diversity of PPARγ forms, describe various ligands and their use in different diseases, and finally focus on the use of PPARγ partial agonists for cancer treatment and provide a good overview of ongoing clinical trials.

After a successful review of PPARβ/δ in the hallmarks of cancer [42], we decided for the current Special Issue to provide a comprehensive analysis of the literature for all PPAR isoforms in relation to the hallmarks of cancer. We describe the known roles of PPARs in cancer cell proliferation, cell death, angiogenesis, invasion and metastasis, replicative immortality, tumor metabolism, and cancer immunity and graphically illustrate the signaling pathways involved therein [43]. Of note, the hallmarks of cancer are a didactic concept, and from a single positive effect on one of the hallmarks, a potential therapeutic effect of PPAR modulation cannot be predicted. Clinical studies and profound retrospective analyses of the available data are required to answer the therapeutic potential of PPAR modulation for cancer.

Mukherjee et al. summarize the role of PPARs and non-coding RNAs in non-alcoholic fatty liver disease (NAFLD) [44]. They introduce the causes and pathology of NAFLD and describe the roles of the different PPAR isoforms in this disease. Afterwards, they describe in detail knowledge about microRNAs, long non-coding RNAs, and circular RNAs in NAFLD and the potential regulation of PPARs by non-coding RNAs. As RNA-based therapies are becoming increasingly focused on, they provide an outlook of how these potential therapies might be used in the future to modify the progression of non-alcoholic fatty liver disease.

Siblini and colleagues explore the influence of methionine needs and the SIRT1/PGC-1α/PPAR-α axis on normal and cancer stem cells [45]. Normal and cancer stem cells share some common features of self-renewal and differentiation capacity. The one-carbon metabolism (OCM) plays an important role in self-renewal and differentiation through its role in the endogenous synthesis of methionine and S-adenosylmethionine (SAM), the universal donor of methyl groups in eukaryotic cells. Stem cells’ reliance on methionine is linked to several mechanisms, including a high methionine flux or low endogenous methionine biosynthesis. The authors highlight the influence of SIRT1 on SAM synthesis and suggest the role of PGC-1α/PPAR-α in impaired stemness produced by methionine deprivation. Of high interest is the potential of methionine restriction in regenerative medicine and cancer treatment.

Guo et al. review the potential roles of PPARs in the fetal origin of adult diseases [46]. The fetal origin of adult disease (FOAD) hypothesis postulates that early events might predispose the development of certain diseases later in life. More than 30 years ago, it was already noted that an increased risk of death from stroke and coronary heart disease in adults was related to a low birth weight [47]. Later, the concept was expanded to different diseases, and it was even reported that transgenerational effects have their origin in early embryos [48]. The roles of PPARs in FOAD have been increasingly appreciated due to their wide variety of biological actions. Exposure to different events in early life has a significant influence on the methylation pattern of PPARs in several organs, which can affect development and health throughout the course of life. In this excellent review, the authors have compiled recent data on the role of PPARs in the fetal origin of different adult diseases and provide potential ways to prevent such diseases in the future. 

In summary, this Special Issue, “The Role of PPARs in Disease II”, represents an excellent collection of original articles which might open up new perspectives for therapeutic interventions and comprehensive up-to-date reviews on several different topics of PPAR signaling in different disease processes. In contrast to earlier descriptions, the roles of PPARs are not limited to metabolic alterations as many different opportunities in neurological, cardiovascular, hepatic diseases, regenerative medicine, and cancer emerge.
